# Sex hormone profiles in men with migraine: a cross-sectional, matched cohort study

**DOI:** 10.3389/fneur.2025.1648017

**Published:** 2025-08-18

**Authors:** Paul Triller, Elisabeth Storch, Lucas H. Overeem, Mira P. Fitzek, Carolin L. Hoehne, Maria Terhart, Kristin S. Lange, Uwe Reuter, Bianca Raffaelli

**Affiliations:** 1 Department of Neurology, Charité – Universitätsmedizin Berlin, corporate member of Freie Universität Berlin and Humboldt-Universität zu Berlin, Berlin, Germany; 2 Junior Clinician Scientist Program, Berlin Institute of Health at Charité (BIH), Berlin, Germany; 3 Universitätsmedizin Greifswald, Greifswald, Germany

**Keywords:** migraine, CGRP, sex hormones, estrogen, progesterone, testosterone, men

## Abstract

**Objective:**

Sex hormones play a key role in migraine pathophysiology, yet their impact in men remains unclear. This study investigates sex hormone profiles and their potential relationship with Calcitonin Gene-Related Peptide (CGRP) in men with episodic migraine.

**Methods:**

We analyzed serum blood levels of sex hormones testosterone, estradiol (E2), progesterone, follicle-stimulating hormone (FSH), luteinizing hormone (LH) and CGRP in age and body mass index (BMI)-matched men with and without migraine.

**Results:**

A total of 120 male participants (*n* = 60 with migraine and *n* = 60 without migraine) completed the study. The mean age was 44.4 ± 14.4 years in migraine group and 44.5 ± 16.2 in the control group. Men with migraine had lower progesterone levels (0.2 nmoL/L, IQR 0.2) and a higher E2 to progesterone (E2/P) ratio (0.33, IQR 0.26) compared to healthy controls (0.5 nmoL/L, IQR 0.2, *p* < 0.001; 0.25, IQR 0.19, *p* < 0.02). Median E2 was 0.09 nmoL/L (IQR 0.03) in migraine patients and 0.12 nmoL/L (IQR 0.04) in controls (*p* = 0.07). There were no significant differences in testosterone, testosterone to E2 (T/E2) ratio, LH and FSH levels. CGRP serum levels did not differ between groups and showed no correlation with sex hormone levels. Subgroup analysis revealed no differences in hormone or CGRP levels between migraine patients with and without aura.

**Discussion:**

Our findings indicate higher progesterone levels and lower E2/P ratios in healthy men compared to those with migraine, suggesting a potential association between sex hormone profiles and migraine in men. These results warrant further investigation into the hormonal modulation of migraine beyond the female population.

## Introduction

Migraine is a prevalent and disabling neurological disorder affecting women approximately three times more often than men (1). The involvement of female sex hormones in migraine pathophysiology is well recognized - particularly the “estradiol withdrawal hypothesis,” which links rapid declines in estradiol levels to migraine attacks (2). However, the influence of sex hormones on migraine in men remains less understood (3). Clinically, men with migraine tend to experience shorter attacks compared to women (4). In some studies, they also report lower pain intensity and less frequent accompanying symptoms such as photophobia, phonophobia, nausea, and vomiting (4, 5). Migraine in men is probably underdiagnosed, partly due to sociocultural factors that discourage seeking medical attention for conditions often perceived as purely feminine (6, 7). The male sex hormone profile is characterized by higher testosterone levels compared to women, alongside lower concentrations of estradiol (E2) and progesterone (8). Testosterone has been suggested to play a protective role in migraine susceptibility due to its anti-inflammatory, neuroprotective, and analgesic properties (9, 10). Some studies have reported lower testosterone levels in men with chronic migraine compared to healthy controls, although findings remain inconsistent, with other studies showing no significant differences (11–13). Beyond testosterone, research on E2 in men with migraine has yielded conflicting results, with one study reporting increased levels and another finding no differences compared to controls (12, 14). Progesterone may play a protective role, and lower levels have been observed in men with migraine (13). Additionally, evidence indicates that fluctuations in the balance between testosterone and estrogen throughout life may contribute to migraine susceptibility in men (14).

Calcitonin gene-related peptide (CGRP) is a key neuropeptide in the pathophysiology of migraine, playing a central role in the initiation of attacks. Elevated CGRP levels have been observed in migraine patients, particularly during attacks and interictal in chronic migraine (15–17). However, findings in episodic migraine (EM) are more variable, with some studies reporting increased CGRP levels while others show no significant differences compared to healthy controls (18). Our previous work in women suggested that sex hormones influence CGRP levels, with higher CGRP concentrations observed in the perimenstrual period in women with EM compared to healthy controls (19). An older study reported lower plasma CGRP levels in men compared to women; however, this study did not account for migraine diagnosis, endocrine disorders, or sex hormone profiles, limiting its interpretability (20). Up to date, the relationship between sex hormones and CGRP regulation in men with migraine remains unclear (3, 12).

In this study, we investigate the interplay between sex hormones, CGRP, and migraine in men. By analyzing sex hormone levels and CGRP plasma concentrations in men with EM and healthy controls, we aim to identify differences in hormonal profiles and CGRP expression that may contribute to migraine susceptibility in men.

## Methods

### Study design and patient selection

This exploratory cross-sectional, matched-cohort study represents an extension of a previously published investigation on the influence of sex hormones on CGRP in women (19). The study was conducted at the Headache Center, Department of Neurology, Charité – Universitätsmedizin Berlin, between August 2020 and January 2024. The study cohort consisted of male patients with EM, with and without aura, and an age-and BMI-matched male control group. Migraine patients were recruited from our outpatient headache clinic, while healthy controls were primarily recruited from university and hospital staff, as well as personal acquaintances, through announcements or direct outreach.

### Inclusion and exclusion criteria

EM with and without aura was defined according to the International Classification of Headache Disorders 3 (ICHD-3) criteria (ICHD-3) (21). We included patients with 3–14 migraine days in the 4 weeks prior to the study visit, as documented in a headache diary. Participants were excluded if they had any other diagnosed primary headache disorder except tension-type headache on fewer than 2 days in the month before screening. Individuals with chronic migraine were excluded. Participants were also excluded if they had ongoing migraine prophylactic treatment, any other neurological disease, or a relevant medical condition requiring drug treatment. Additionally, undergoing hormonal therapy or a history of hypogonadism led to exclusion from the study.

### Study procedures

The study consisted of a single visit, which included an initial eligibility screening followed by sample collection for those meeting all criteria. During screening, potential participants underwent a medical history interview and a physical examination, including assessment of vital signs. All migraine patients at our center are required to maintain a headache diary, which was reviewed to verify headache frequency in the previous 4 weeks. Additionally, male participants with migraine completed the Headache Impact Test-6 (HIT-6), which is a validated questionnaire assessing headache-related burden, including pain severity, daily limitations, and emotional distress. Scores range from 36 to 78, with higher values indicating greater impairment (22). Eligible participants proceeded to sample collection, which was conducted under non-fasting conditions following standardized protocols. Migraine patients were required to be in an interictal phase, meaning they had to be free of migraine symptoms and had not taken any pain medication for at least 12 h before and after the visit. To confirm interictal status, study staff contacted migraine patients 24 h post-visit to assess whether they remained migraine-free. If a migraine attack occurred in the 12 h after the visit, the visit was repeated to ensure data collection in an interictal phase. For hormone analysis, a 5-mL blood sample was collected in serum tubes (BD Vacutainer) and sent to our partner laboratory, Labor Berlin, Charité Vivantes GmbH for the quantification of E2, progesterone, testosterone, luteinizing hormone (LH), and follicle-stimulating hormone (FSH) using an electrochemiluminescence immunoassay. After analyzing data from the first 60 participants, the measurement of free testosterone was incorporated for the subsequent 60 participants to provide a more comprehensive hormonal profile. Therefore, values for free testosterone are available for 60 participants, while data for all other hormones are available for the full cohort of 120 participants.

**Figure 1:**
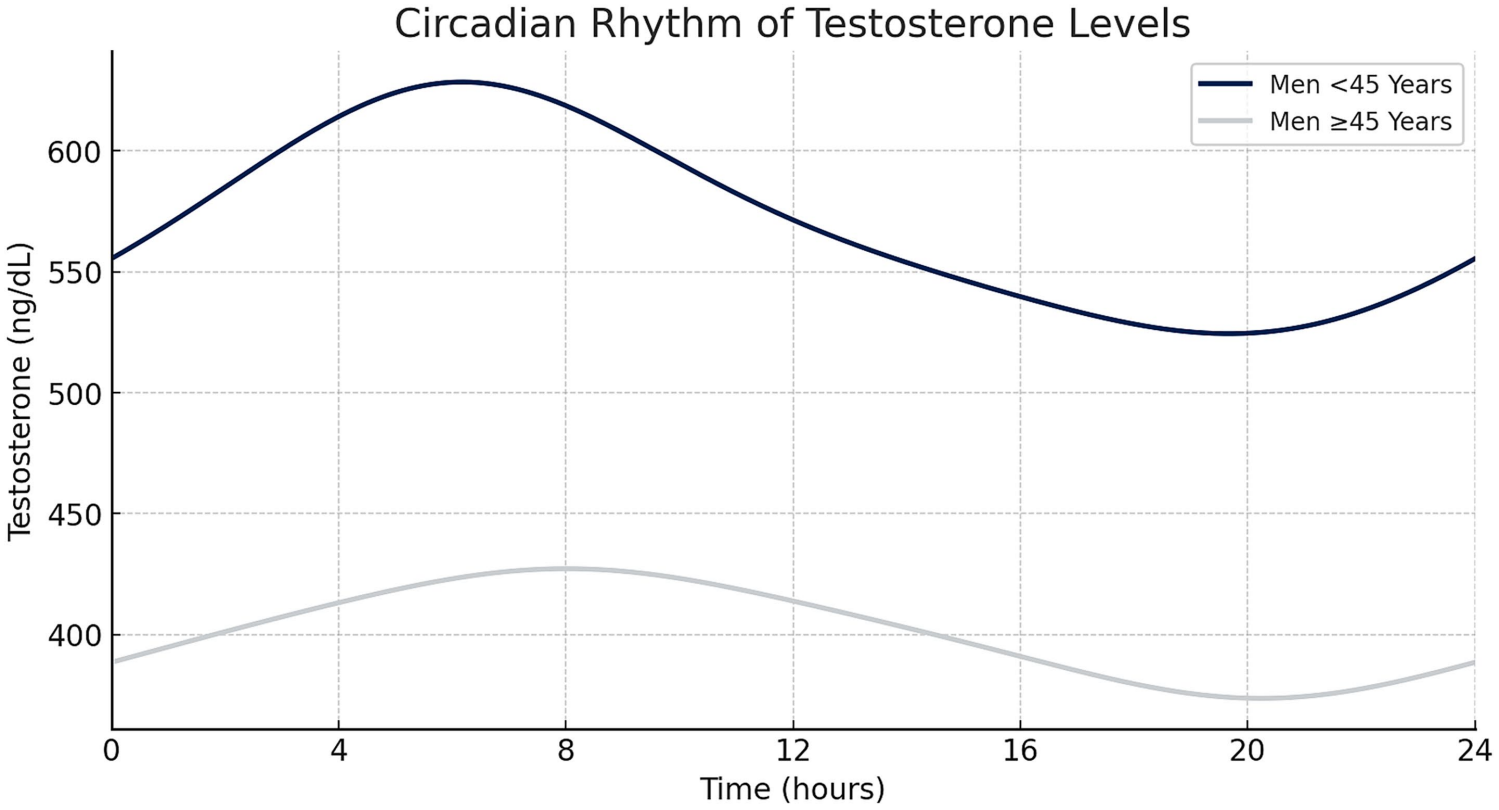
Circadian testosterone fluctuations over 24 h in men aged <45 and ≥45 years, demonstrating an early morning peak and subsequent decline throughout the day (created with Microsoft Excel).

To account for circadian fluctuations in testosterone levels, values were adjusted to a standardized 6 p.m. reference point using a model derived from a cohort study by Gupta et al. This model differentiates between men older and younger than 45 years, as illustrated in Figure 1, and is based on:

$$\begin{array}{l} \text{Cpop}\;(t)=A0+(\text{Cpeak}-A0\;)\cdot \mathrm{e\sigma}\cdot (\cos(\theta (t-\text{Tpeak}\;))-1)\\ +(\text{Cnadir}-A0\;)\cdot \mathrm{e\sigma}\cdot (\cos(\theta (t-\text{Tnadir}\;))-1) \end{array}$$

Where Cpop(t) represents testosterone levels at time t, A0 the baseline, Cpeak and Cnadir the peak and nadir levels, and Tpeak and Tnadir their respective time points (23).

For CGRP analysis, blood samples were collected in precooled 5-mL EDTA tubes (BD Vacutainer) with 200 μL aprotinin added to prevent from early degradation of CGRP through proteolysis. Samples were immediately centrifuged at −6°C for 15 min at 2,000 rpm, and the plasma was transferred into 1.5-mL polypropylene tubes (Eppendorf, Hamburg, Germany). Plasma samples were stored at −80°C until analysis. CGRP levels were quantified using a commercial enzyme immunoassay (EIA) kit (Bertin Bioreagent, Montigny le Bretonneux, France) with a detection range of 8–1,000 pg./mL and a sensitivity of 2 pg./mL. This EIA detects all human CGRP isoforms and is validated for use in blood, plasma, serum, cerebrospinal fluid, and nervous tissue extract (24). To minimize variability, samples from migraine and control groups were analyzed in parallel, maintaining similar group proportions per EIA kit and adhering to the manufacturer’s instructions.

### Endpoints

The primary objective of this study was to investigate sex hormone profiles in men with migraine compared to healthy controls. Specifically, we examined differences in the levels of LH, FSH, total testosterone, circadian-adjusted testosterone (Tc), free testosterone (Tf), E2, and progesterone between the two groups. Additionally, we analyzed the ratios of total testosterone, Tc, and Tf to E2, as well as the ratio of E2 to progesterone. Furthermore, an exploratory subgroup analysis was performed to compare hormone concentrations between migraine patients with and without aura.

As an exploratory endpoint, we assessed CGRP levels in men with migraine versus healthy controls and explored potential correlations between CGRP levels, sex hormone concentrations, and clinical or demographic variables, including monthly migraine days (MMD), attack duration, intensity, and HIT-6 scores. We also explored whether CGRP levels differed between migraine patients with and without aura or showed distinct correlations within these subgroups.

### Statistical analyses

A formal *a priori* power calculation was not conducted due to the limited availability of comparable data at the study’s outset. To mitigate this, we doubled the sample size used in a previous study (19) and included 60 participants to increase statistical power and ensure more robust analyses. Demographic, clinical, and laboratory data were summarized using descriptive statistics. Numerical variables were presented as means with standard deviations (SD) or medians with interquartile ranges (IQR), depending on data distribution. Categorical variables were reported as frequencies and percentages. Normal distribution was assessed using visual inspection of histograms and the Shapiro–Wilk test. To compare continuous variables between migraine patients and controls, Student’s *t*-tests were used for clinical and demographic variables that followed normal distribution (e.g., heart frequency, blood pressure). For non-normally distributed variables including all laboratory data (sex hormones and CGRP levels) the Mann–Whitney U test was used. Correlations between CGRP levels, sex hormones were evaluated using Spearman’s rank correlation coefficient. All hypothesis tests were two-sided, with a significance threshold of *p* < 0.05. Missing data were handled by complete case analysis. Participants with missing values for a specific variable were excluded from related analyses but remained included in other analyses. All participants met inclusion criteria, and missing data did not affect eligibility or group assignment. Statistical analyses were conducted using IBM SPSS Statistics, version 29.0 (IBM, Armonk, NY, USA).

### Standard protocol approvals, registrations, and patient consents

The Charité Ethical Committee approved the study protocol (EA1/004/20). All participants consented in writing after receiving study information

### Data availability

Data not provided in the article because of space limitations may be shared (anonymized) at the request of any qualified investigator for purposes of replicating procedures and results.

### Dis****

In this study, we specifically focused on male participants who identified as men. It is important to note that the results of this study may not necessarily be generalizable to all individuals who identify as men, as gender identity and biological sex may influence outcomes in different ways.

## Results

### Patient characteristics

We included *n* = 120 male participants in the study visiting our tertiary headache center between August 2020 and January 2024. All participants completed the study protocol, with *n* = 60 in the migraine group and *n* = 60 in the control group. The demographic characteristics did not differ between groups (see Table 1). The average age of migraine patients was 44.4 (±14.4) years, with a disease duration of 21.4 (±16.3) years. Twenty-four patients (40%) reported a history of migraine aura, defined as at least two aura episodes over their lifetime. Migraine patients had an average of 6.1 (±3.3) monthly migraine days (MMDs) in the month prior to inclusion, with a mean pain intensity of 6.5 (±1.6) on the numerical rating scale (NRS), and an average attack duration of 21.4 (±16.3) hours.

**Table 1:** Description of study population.

	Migraine mean (SD) or *n* (%)	*n*	Control mean (SD) or *n* (%)	*n*	*p*
Age	44.4 (±14.4)	60	44.5 (±16.2)	60	0.98
Aura	24 (41.3)	58			
MMD	6.1 (±3.3)	58			
Attack duration (h)	21.4 (±16.3)	59			
Pain intensity (NRS)	6.5 (±1.6)	58			
Disease duration (years)	18.2 (±12.1)	58			
HIT-6	59.8 (±5.9)	57			
BMI	24.7 (±3.8)	59	25.1 (±3.6)	60	0.58
BP systolic (mmHg)	132.8 (±12.7)	54	132.8 (±18.6)	57	0.99
BP diastolic (mmHg)	82.5 (±10.7)	53	75.3 (±11.4)	56	<0.001
Heart rate (bpm)	74.3 (±12.8)	54	79.0 (±15.3)	57	0.08

MMD, monthly migraine days; NRS, numeric rating scale; BMI, body mass index; BP, blood pressure.

The average blood pressure in migraine patients was 132.8 (±12.7) /82.5 (±10.7) mmHg, compared to 132.8 (±18.6) /75.3 (±11.4) mmHg in the control group, with a significant difference in diastolic values (*p* < 0.001).

### Sex hormones

#### Progesterone and estradiol

**Figure 2:**
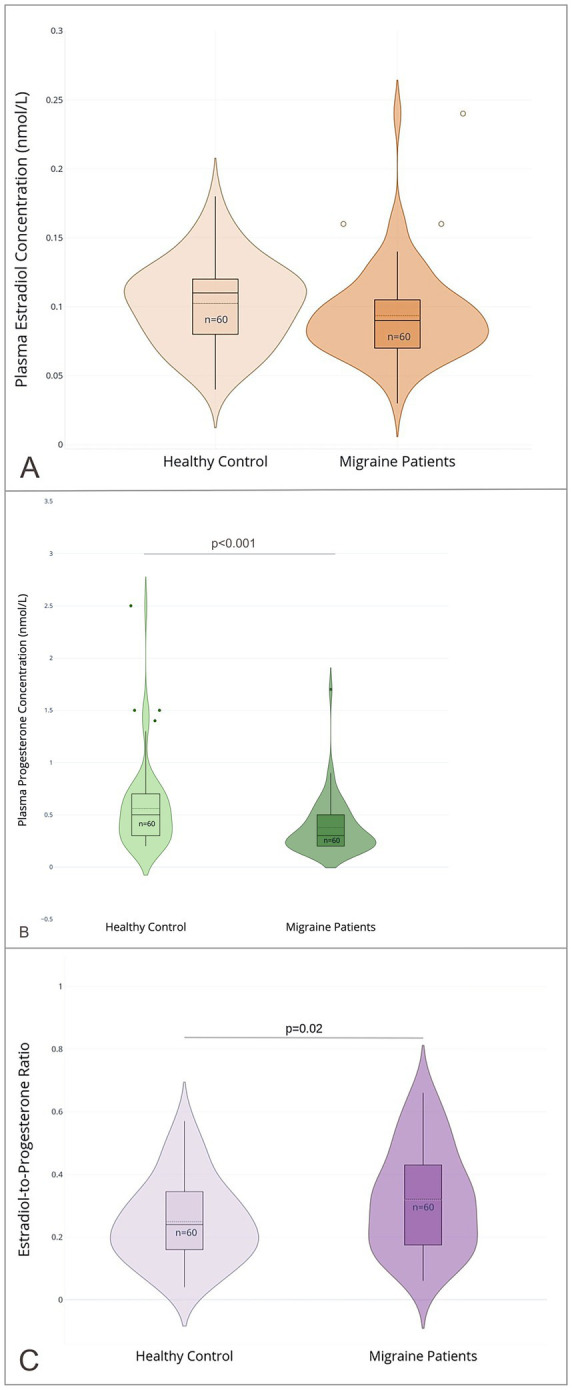
Sex hormone profiles in male migraine patients and healthy controls. **(A)** Plasma estradiol (E2) concentrations (nmol/L); **(B)** plasma progesterone (P) concentrations (nmol/L); **(C)** the estradiol-to-progesterone ratio (E2/P); in male migraine patients (*n* = 60) and healthy controls (*n* = 60). Violin plots depict the distribution of values with overlaid boxplots showing median and interquartile range (IQR). Outliers are displayed as individual points (created with Plotly Technologies Inc.).

The migraine group had a significantly lower serum progesterone concentration, with a median of 0.2 nmol/L (IQR 0.2), compared to 0.5 nmol/L (IQR 0.3) in the healthy control group (p < 0.001). Similarly, serum E2 concentration was lower in the migraine group (0.09 nmol/L, IQR 0.03) than in the control group (0.12 nmol/L, IQR 0.04; *p* = 0.07), though this difference was not statistically significant. This pattern was also reflected in the E2/P ratio, which was significantly higher in the migraine group (0.33, IQR 0.26) compared to the control group (0.25, IQR 0.19; *p* = 0.02). Results are shown in Figure 2 and Supplementary Table 2. No significant correlations were found between progesterone, E2, or their respective ratios and clinical outcomes such as migraine days, pain intensity, or aura frequency (See Supplementary Table 6).

#### Testosterone

**Figure 3:**
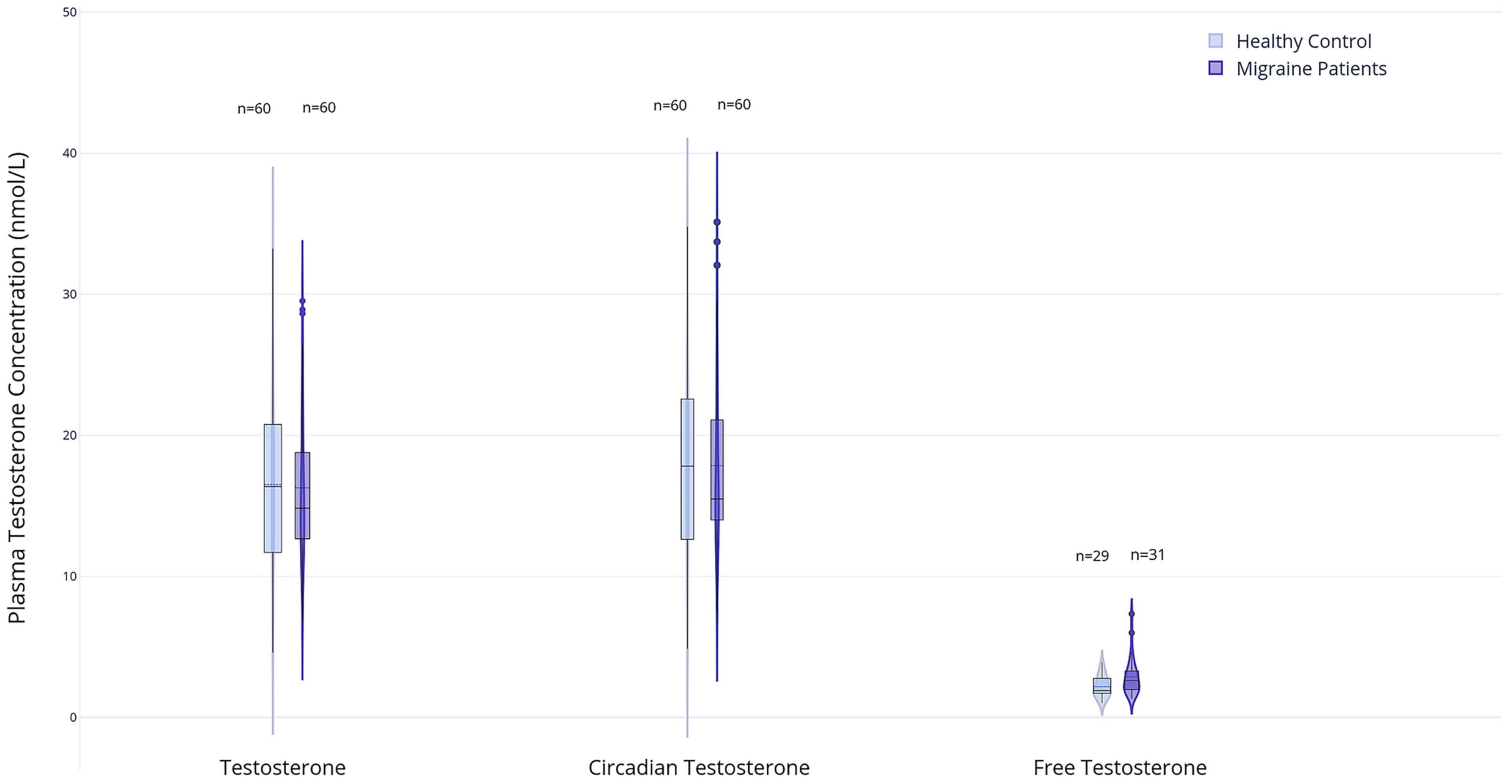
Box and violin plots represent plasma levels of three testosterone measures in male migraine patients (*n* = 60) and healthy controls (*n* = 60): total testosterone (T), circadian testosterone (Tc), free testosterone (Tf). For Tf, *n* = 29 (healthy control) and *n* = 31 (migraine patients). Data distributions are visualized using violin plots; boxplots show medians and interquartile ranges (created with Plotly Technologies Inc.).

**Figure 4:**
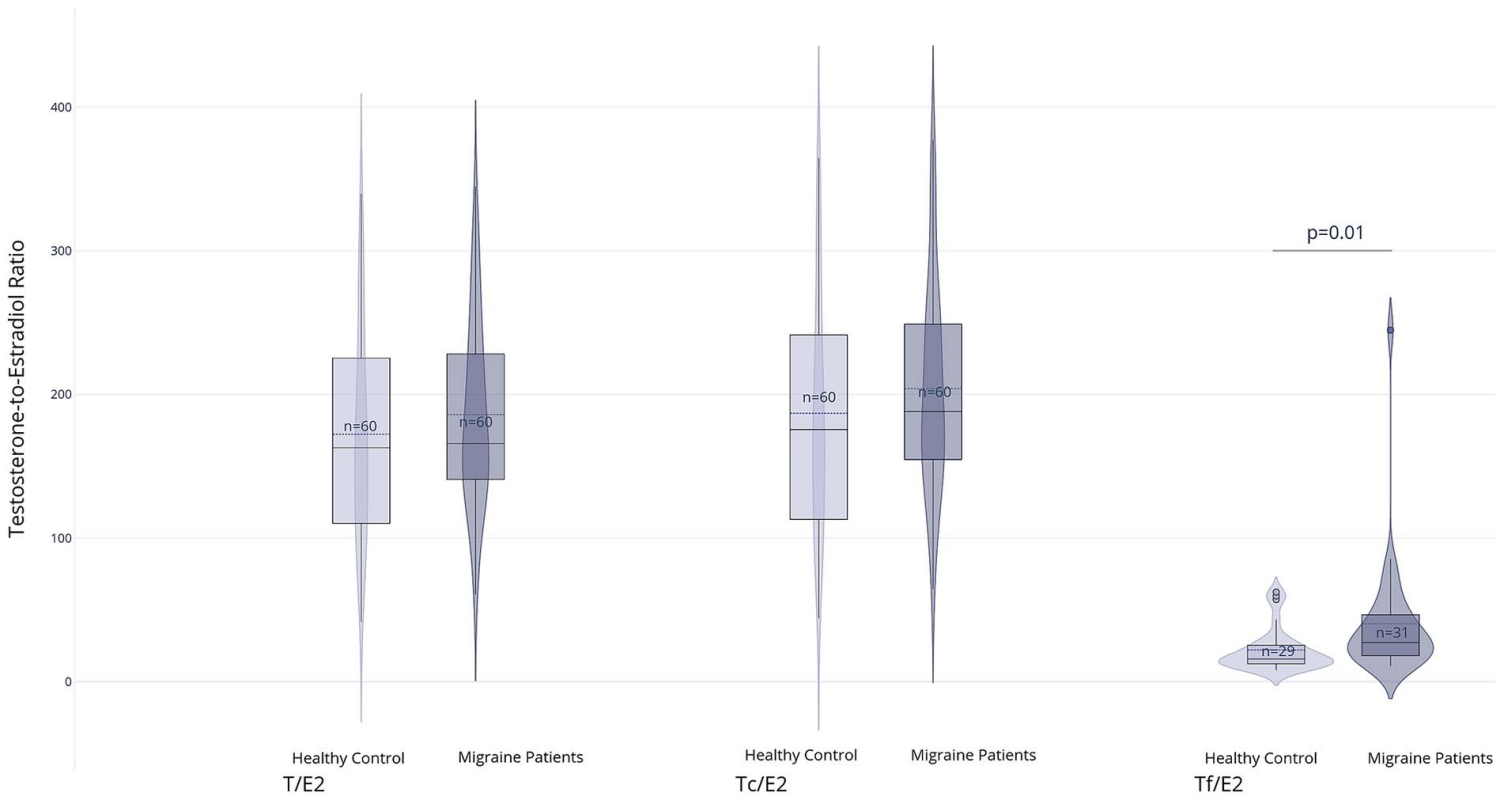
Group comparisons of calculated ratios between testosterone and estradiol: T/E2: Total testosterone to estradiol ratio, Tc/E2: Circadian testosterone to estradiol ratio, Tf/E2: Free testosterone to estradiol ratio. For the Tf/E2 ratio, values were available for *n* = 29 (healthy control) and *n* = 31 (migraine patients) (created with Plotly Technologies Inc.).

There were no significant differences in serum testosterone levels, including time-corrected values, between the migraine and control groups (T: 14.0 nmol/L, IQR 4.7 vs. 15.0 nmol/L, IQR 8.8; *p* = 0.64; Tc: 14.8 nmol/L, IQR 4.0 vs. 15.6 nmol/L, IQR 9.7; *p* = 0.68). Similarly, Tf levels did not differ significantly between groups (0.20 nmol/L, IQR 0.10 in both groups; *p* = 0.96). However, the Tf/E2 ratio was significantly higher in the migraine group (2.6, IQR 1.4) than in the control group (1.9, IQR 1.1; *p* = 0.01), whereas the total T/E2 ratio and the Tc/E2 ratio showed no significant differences (T/E2: 154.4, IQR 111.4 vs. 117.8, IQR 85.2; *p* = 0.27 and Tc/E2: 168.0, IQR 113.0 vs. 122.9, IQR 85.5; *p* = 0.19). Results are displayed in Figures 3, 4 and Supplementary Table 2.

### Analysis by aura status

In the subgroup analysis of migraine patients with (*n* = 24) and without aura (*n* = 34), no statistically significant differences were observed for testosterone (16.4 nmol/L, IQR 8.2 vs. 14.3 nmol/L, IQR 4.74; *p* = 0.301), Tc (17.6 nmol/L, IQR 6.2 vs. 15.3 nmol/L, IQR 4.4; *p* = 0.368), or Tf (both groups: 0.23 nmol/L, IQR 0.08 *p* = 0.804).

Similarly, E2 (0.09 nmol/L, IQR 0.02 in both groups; *p* = 0.283), P (0.3, IQR 0.3 in both groups; *p* = 0.68), and all calculated hormone ratios (E2/P, T/E2, Tc/E2, Tf/E2) did not differ significantly between the two subgroups. Median values and interquartile ranges are provided in Supplementary Table 3.

### CGRP

**Figure 5:**
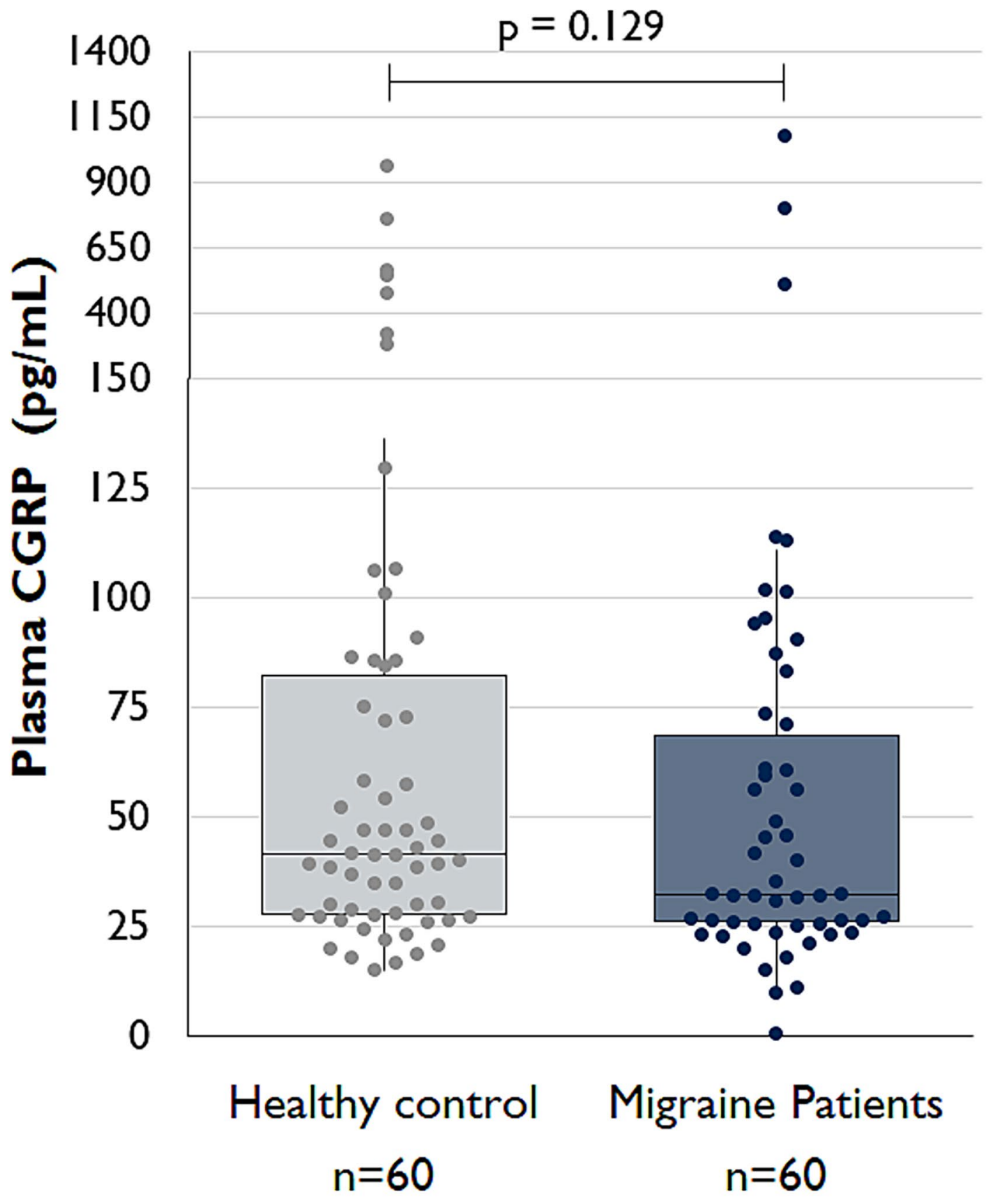
Comparison of interictal serum CGRP levels between migraine patients and controls (Boxplots created with Microsoft Excel).

Interictal CGRP serum concentrations did not differ significantly between groups, with a median of 32.2 pg./mL (IQR 45.3) in the migraine group and 41.6 pg./mL (IQR 57.7) in the control group (*p* = 0.129; see Figure 5).

Additionally, no significant correlations were observed between CGRP levels and E2, progesterone, testosterone, Tc, Tf, LH, or FSH, nor with clinical parameters such as migraine days, pain intensity, or aura frequency (see Supplementary Tables 4, 6).

In a subgroup analysis, CGRP concentrations also did not differ between migraine patients with aura 26.7 pg./mL (IQR 32.7) and those without aura 32.4 pg./mL (IQR 58.8); *p* = 0.072. There was no significant correlation between CGRP levels and sex hormones or clinical parameters within migraine with and without aura subgroups (Supplementary Tables 3, 5, 7).

## Discussion

In this cohort of male participants, migraine patients exhibited significantly lower serum progesterone levels and higher E2/P and Tf/E2 ratios compared to healthy controls. No significant differences were observed in other sex hormone levels, their respective ratios, or interictal serum CGRP concentrations between the groups. Furthermore, no significant correlations were found between sex hormones or their ratios, and clinical outcomes such as migraine days, pain intensity, or aura frequency.

Historically, studies from the 1970s suggested that migraine was triggered primarily by estrogen withdrawal rather than progesterone fluctuations (25) and that progesterone administration did not provide a protective effect (26). However, more recent evidence challenges this view. Progesterone appears to suppress nociception in the trigeminal pathway, likely through its conversion to allopregnanolone, a neurosteroid that enhances GABAergic inhibitory signaling (27–29). In our study, the observation that male migraine patients had lower serum progesterone levels than healthy men supports the hypothesis that reduced progesterone may be associated with increased migraine susceptibility. We further propose that a protective hormonal profile - characterized by relatively high and stable levels of both progesterone and estrogen - may exist independently of sex. This pattern is observed in our study’s healthy male controls and in women during hormonal phases less susceptible to migraine, such as the late follicular phase and pregnancy (30). Clinical observations in women using progestin-only contraception support this concept, as continuous progestin administration seems to be associated with a reduction in migraine frequency (31). Supporting our findings, a subgroup analysis of a Chinese cohort study similarly reported higher serum progesterone levels in healthy male controls compared to men with migraine, along with a negative correlation between progesterone levels and headache burden in the migraine group (13). In contrast, our study found no significant correlations between sex hormones and clinical headache features, highlighting the complexity and multifactorial nature of hormonal influences on migraine and the potential variability across populations and study designs.

While progesterone may exert protective effects through modulation of trigeminovascular nociception, the role of testosterone and the Tf/E2 ratio in migraine remains less clear. Previous studies have yielded conflicting results: Shields et al. reported lower total testosterone levels in men with chronic migraine compared to healthy controls, while Van Oosterhout et al. found that men with EM had lower interictal testosterone levels and a reduced Tf/E2 ratio (11, 12). The differences between our study and previous investigations may be attributed to methodological variations in hormone measurement and participant selection. While Shields et al. exclusively analyzed total testosterone in men with chronic migraine, our study assessed total, circadian-corrected, and free testosterone levels, as well as various hormonal ratios, specifically in the context of EM. Importantly, our study ensured interictal sampling, with participants being headache-and medication-free for at least 12 h before and after blood collection. Differences in sampling protocols, particularly regarding the timing and standardization of hormone assessments, may have contributed to the variation in results (32–34).

Regarding CGRP, our study found no significant differences in interictal serum levels between migraine patients and healthy controls, nor did we observe correlations between sex hormones and CGRP. The existing literature on CGRP concentrations in peripheral blood is extensive yet contradictory. While some studies report no differences in interictal CGRP levels in episodic migraine patients, others suggest changes only in chronic migraine and during ictal phases (17, 35, 36). Thus, the role of CGRP as an interictal biomarker of migraine remains to be clarified.

To our knowledge, this is the first study to investigate sex hormone and CGRP concentrations in a cohort of male patients with episodic migraine stratified by aura status. We found no significant differences in hormone or CGRP levels between patients with and without aura. In addition, no relevant correlations were observed between neuropeptide or hormone concentrations and demographic or clinical variables such as age, BMI, migraine frequency, or disability scores. In contrast, a recent case–control study in a mixed-gender population (predominantly female) reported significantly higher interictal concentrations of CGRP, VIP, and PACAP-38 in patients with migraine with aura compared to those without aura and healthy controls Reference. Moreover, neuropeptide levels, particularly CGRP, were more strongly correlated with attack frequency and headache-related disability in patients with aura, suggesting a possible subtype-specific pathophysiological profile (37).

However, it is important to note that most hormonal research in migraine has focused on cyclical variation, menstrual migraine, or the effects of hormonal contraception, without further stratification by aura subtype (19, 38). Our findings provide novel evidence from a male sample and highlight the need for future studies to explore potential sex-and subtype-specific endocrine mechanisms in migraine pathophysiology.

In addition to hormonal differences, we observed a significant difference in diastolic blood pressure, with lower values in the control group. The observed difference in blood pressure is noteworthy and warrants further consideration. Elevated diastolic blood pressure in migraine patients has been linked to an increased risk of cardiovascular comorbidities. A large population-based study in Iceland reported that for each one standard deviation increase in diastolic blood pressure, the probability of having migraine increased by 14% in men (39). Emerging evidence suggest that progesterone may influence sympathetic activity, potentially affecting vascular tone and blood pressure regulation (40, 41). Lower levels of progesterone in migraine men could contribute to elevated diastolic blood pressure. Our findings may suggest a broader connection between migraine, hormonal regulation and cardiovascular health (3). This is the first study to investigate sex hormones and CGRP in male migraine patients using a relatively large cohort of 60 patients and 60 age-and BMI-matched healthy controls. Our sample was carefully selected to minimize confounding factors, and hormone analyses were conducted in a central laboratory blinded to group allocation to ensure measurement accuracy. However, several limitations should be acknowledged. Current measurement techniques and sample collection protocols may lack the sensitivity to detect subtle variations in CGRP and hormone levels. fT measurements using chemiluminescence immunoassays have known limitations in accuracy, highlighting the need for more sophisticated methods such as equilibrium dialysis or ultrafiltration. Similarly, at the low E2 levels typically found in men, mass spectrometry-based assays offer superior precision compared to standard immunoassays. Although not always available, applying these advanced methods in future studies could help to validate and expand upon our findings (42, 43). Additionally, blood samples were collected under non-fasting conditions between 9 a.m. and 4 p.m., rather than during the early morning peak for testosterone, requiring age-based corrections for circadian variations (23, 44). Although LH and FSH exhibit pulsatile secretion and can show short-term fluctuations, previous studies have demonstrated that diurnal variations in LH and FSH during typical clinical sampling hours are minimal and generally not considered clinically relevant for standard assessments in men (45). Nocturnal variations are more pronounced than daytime fluctuations, and dynamic or serial sampling would be required to capture these patterns (46). Due to the lack of validated correction functions in the literature, we did not adjust for circadian fluctuations in LH and FSH (44) and opted for a pragmatic approach in line with standard clinical practice, correcting only for testosterone.

Another limitation is the relatively short interictal period, defined as at least 24 h without migraine or use of acute medication. This duration is shorter than in similar studies and that migraine-related pathophysiological changes can occur in pro-and postdromal phases lasting from several hours up to several days (47). Extending the interictal period definition to more than 24 h, while scientifically desirable, poses practical challenges for participant recruitment and adherence, particularly in episodic migraine populations where attacks are frequent and unpredictable. We acknowledge that this limitation may have introduced some variability into the hormonal and biomarker measurements. Nonetheless, the use of a 24-h window allowed for feasible and consistent data collection across participants while reflecting real-world clinical settings. Future studies could address this limitation by incorporating longer headache-free intervals or even prospective monitoring with serial sampling to better delineate the interictal, preictal, and postictal phases of migraine with respect to hormonal dynamics.

Furthermore, there were some missing data in the demographic, clinical, and headache-related variables, which may limit the generalizability and completeness of the analyses. We addressed this by using complete case analysis, excluding participants only from analyses where relevant data were missing while retaining them in all other applicable analyses. Although this approach preserves available data, it may have reduced statistical power for some comparisons.

## Conclusion

This study showed significantly lower serum progesterone levels and higher E2/P and Tf/E2 ratios in migraine patients compared to healthy controls. These findings support the hypothesis that progesterone may have a protective effect against migraine, while the balance between testosterone and estrogen, rather than absolute hormone levels, may influence migraine susceptibility. In contrast to previous reports, no significant differences in CGRP levels were observed between groups, highlighting the ongoing challenges in capturing interictal CGRP fluctuations. Future research should consider a longitudinal design to better explore the temporal dynamics of hormonal fluctuations in relation to migraine attacks.

## Data Availability

The raw data supporting the conclusions of this article will be made available by the authors, without undue reservation.

## Glossary

BMI: Body Mass Index
CGRP: Calcitonin Gene-Related Peptide
CM: Chronic Migraine
E2: Estradiol
E2/P: Estradiol-to-Progesterone Ratio
EDTA: Ethylenediaminetetraacetic Acid
ELISA: Enzyme-Linked Immunosorbent Assay
EM: Episodic Migraine
FSH: Follicle-Stimulating Hormone
HIT-6: Headache Impact Test-6
ICHD-3: International Classification of Headache Disorders, 3rd Edition
IQR: Interquartile Range
LH: Luteinizing Hormone
MMD: Monthly Migraine Days
PACAP: Pituitary Adenylate Cyclase-Activating Polypeptide
BP: Blood Pressure (Systolic/Diastolic)
SD: Standard Deviation
SHBG: Sex Hormone-Binding Globulin
T/E2: Testosterone-to-Estradiol Ratio
Tc: Corrected Testosterone
Tf: Free Testosterone
Tf/E2: Free Testosterone-to-Estradiol Ratio
NRS: Numeric Rating Scale
