# Correction: Sex hormone profiles in men with migraine: a cross-sectional, matched cohort study

**DOI:** 10.3389/fneur.2025.1765206

**Published:** 2026-01-12

**Authors:** Paul Triller, Elisabeth Storch, Lucas H. Overeem, Mira P. Fitzek, Carolin L. Hoehne, Maria Terhart, Kristin S. Lange, Uwe Reuter, Bianca Raffaelli

**Affiliations:** 1 Department of Neurology, Charité – Universitätsmedizin Berlin, corporate member of Freie Universität Berlin and Humboldt-Universität zu Berlin, Berlin, Germany; 2 Junior Clinician Scientist Program, Berlin Institute of Health at Charité (BIH), Berlin, Germany; 3 Universitätsmedizin Greifswald, Greifswald, Germany

**Keywords:** migraine, CGRP, sex hormones, estrogen, progesterone, testosterone, men

There was a mistake in Figure 2B as published. During the creation of the migraine figure, parts of the dataset were not transferred correctly into the violin/box-plot layer, resulting in an incorrect graphical representation of the migraine group. The numerical values, medians, IQRs, and statistical analyses reported in the article are correct; the error affects only the graphical representation. The corrected Figure 2B appears below.

**Figure 2:**
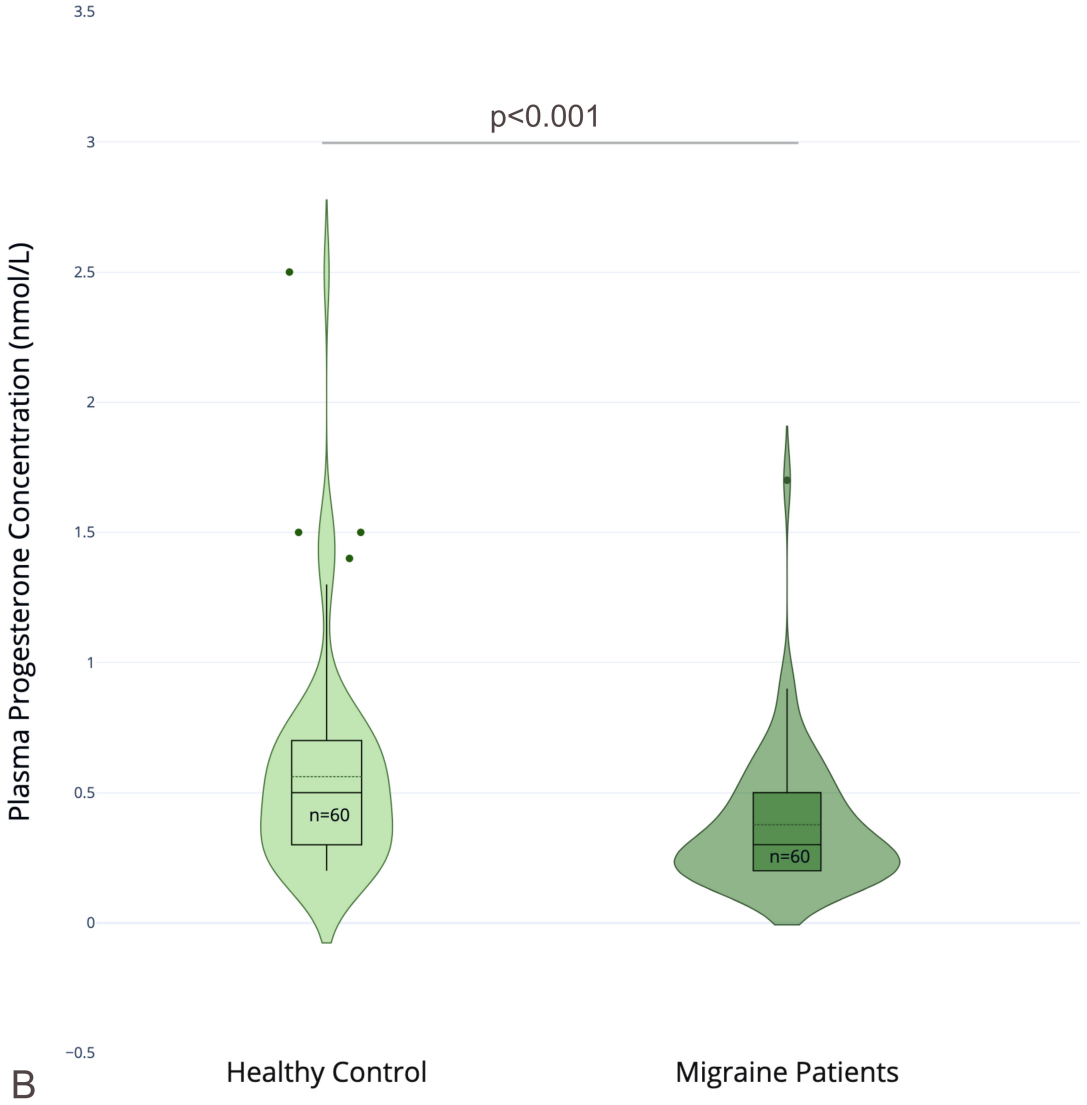
Sex hormone profiles in male migraine patients and healthy controls. **(A)** Plasma estradiol (E2) concentrations (nmol/L); **(B)** plasma progesterone (P) concentrations (nmol/L); **(C)** the estradiol-to-progesterone ratio (E2/P); in male migraine patients (*n* = 60) and healthy controls (*n* = 60). Violin plots depict the distribution of values with overlaid boxplots showing median and interquartile range (IQR). Outliers are displayed as individual points (created with Plotly Technologies Inc.).

The original version of this article has been updated.

