# Supplementary material for: Sex hormone profiles in men with migraine: a cross-sectional, matched cohort study
Source: Front Neurol. 2025 Aug 18;16:1648017. doi: 10.3389/fneur.2025.1648017 (PMC12399798; doi:10.3389/fneur.2025.1648017)
Supplement: Supplementary file 1 [file Table_1.DOCX]

**Electronic Supplementary Material**

**Title:** Sex hormone profiles in men with migraine: A cross-sectional, matched cohort study

**Journal:** Frontiers in Neurology

**Authors**: Paul Triller^†^, Elisabeth Storch^†^, Lucas H. Overeem, Mira P. Fitzek, Carolin L. Hoehne, Maria Terhart, Kristin S. Lange, Uwe Reuter, Bianca Raffaelli

**Corresponding author**: Dr. med. Bianca Raffaelli

**Email:** bianca.raffaelli@charite.de

**Affiliation**: Department of Neurology, Charité – Universitätsmedizin Berlin, Charitéplatz 1, 10117 Berlin, Germany

† contributed equally to this work

**Table 2 Concentrations of sex hormones**

|  | **Migraine**  median (IQR) | | n | **Control**  median (IQR) | n | p |
| --- | --- | --- | --- | --- | --- | --- |
| Progesterone (nmol/l) | | 0.2 (0.2) | 60 | 0.5 (0.3) | 60 | <0.001 |
| E2 (nmol/l) | | 0.09 (0.03) | 60 | 0.12 (0.04) | 60 | 0.07 |
| E2/P Ratio | | 0.33 (0.26) | 60 | 0.25 (0.19) | 60 | 0.02 |
| T(nmol/l) | | 14 (4.7) | 60 | 15 (8.8) | 60 | 0.64 |
| Tc(nmol/l) | | 14.8 (4) | 60 | 15.6 (9.7) | 60 | 0.68 |
| Tf(nmol/l) | | 0.2 (0.1) | 31 | 0.2 (0.1) | 29 | 0.96 |
| T/E2 Ratio | | 154.4 (111.4) | 60 | 117.8 (85.2) | 60 | 0.27 |
| Tc/E2 Ratio | | 168 (113) | 60 | 122.9 (85.5) | 60 | 0.19 |
| Tf/E2 Ratio | | 2.6 (1.4) | 31 | 1.9 (1.1) | 29 | 0.01 |
| LH (U/l) | | 5.2 (3.0) | 60 | 5.5 (3.8) | 60 | 0.11 |
| FSH (U/l) | | 4.8 (2.9) | 60 | 5.4 (3.4) | 60 | 0.24 |

E2 - estradiol, T - testosterone, Tc – circadian testosterone, Tf – free testosterone, LH - luteinizing hormone, FSH - follicle-stimulating hormone

**Table 3 Concentrations of sex hormones and CGRP by aura status**

|  | **Migraine with Aura**  median (IQR) | | n | **Migraine without**  **Aura**  median (IQR) | n | p |
| --- | --- | --- | --- | --- | --- | --- |
| Progesterone (nmol/l) | | 0.3 (0.3) | 24 | 0.3 (0.3) | 34 | 0.93 |
| E2 (nmol/l) | | 0.09 (0.02) | 24 | 0.09 (0.02) | 34 | 0.28 |
| E2/P Ratio | | 0.33 (0.26) | 24 | 0.25 (0.19) | 34 | 0.86 |
| T(nmol/l) | | 16.4 (8.2) | 24 | 14.3 (4.74) | 34 | 0.30 |
| Tc(nmol/l) | | 17.6 (6.2) | 24 | 15.3 (4.4) | 34 | 0.36 |
| Tf(nmol/l) | | 0.2 (0.08) | 11 | 0.2 (0.08) | 20 | 0.80 |
| T/E2 Ratio | | 205.5 (123.1) | 24 | 167.1 (70.1) | 34 | 0.07 |
| Tc/E2 Ratio | | 234.8 (144.2) | 24 | 181.4 (75.6) | 34 | 0.06 |
| Tf/E2 Ratio | | 2.7 (1.8) | 11 | 2.6 (1.2) | 20 | 0.32 |
| LH (U/l) | | 4.4 (2.3) | 24 | 5.4 (3.4) | 34 | 0.37 |
| FSH (U/l) | | 3.6 (3.2) | 24 | 4.1 (3.5) | 34 | 0.49 |
| CGRP(pg/ml)) | | 26.7 (32.7) | 24 | 32.4 (58.8) | 34 | 0.07 |

CGRP- Calcitonin gene-related peptide; E2 - estradiol, T - testosterone, Tc – circadian testosterone, Tf – free testosterone, LH - luteinizing hormone, FSH - follicle-stimulating hormone

**Table 4 Correlations between sex hormones and CGRP**

|  |  | **CGRP** | **Tc** | **Tf** | **E2** | **Progesteron** | **LH** | **FSH** |
| --- | --- | --- | --- | --- | --- | --- | --- | --- |
| **CGRP** | Spearman-Rho | 1 | -015 | -0.16 | 0.07 | -0.22 | -0.03 | 0.1 |
|  | p |  | 0.26 | 0.39 | 0.96 | 0.10 | 0.80 | 0.46 |
|  | n | 60 | 60 | 31 | 60 | 60 | 60 | 60 |

CGRP- Calcitonin gene-related peptide; E2 - estradiol, T - testosterone, Tc – circadian testosterone, Tf – free testosterone, LH - luteinizing hormone, FSH - follicle-stimulating hormone

**Table 5 Correlations between sex hormones and CGRP by Migraine with and without Aura**

| **With Aura** |  | **CGRP** | **Tc** | **Tf** | **E2** | **Progesteron** | **LH** | **FSH** |
| --- | --- | --- | --- | --- | --- | --- | --- | --- |
| **CGRP** | Spearman-Rho | 1 | -0.24 | -0.26 | 0.05 | -0.11 | 0.02 | 0.19 |
|  | p |  | 0.28 | 0.43 | 0.84 | 0.63 | 0.92 | 0.39 |
|  | n | 24 | 24 | 11 | 24 | 24 | 24 | 24 |

| **Without Aura** |  | **CGRP** | **Tc** | **Tf** | **E2** | **Progesteron** | **LH** | **FSH** |
| --- | --- | --- | --- | --- | --- | --- | --- | --- |
| **CGRP** | Spearman-Rho | 1 | -0.63 | 0.01 | -0.08 | -0.25 | -0.09 | 0.05 |
|  | p |  | 0.72 | 0.96 | 0.63 | 0.15 | 0.59 | 0.79 |
|  | n | 34 | 34 | 20 | 34 | 34 | 34 | 34 |

CGRP- Calcitonin gene-related peptide; E2 - estradiol, T - testosterone, Tc – circadian testosterone, Tf – free testosterone, LH - luteinizing hormone, FSH - follicle-stimulating hormone

**Table 6 Correlations between CGRP, sex hormones and clinical parameters**

|  |  | **CGRP** | **Tc** | **E2** | **Progesteron** | **E2/P** |
| --- | --- | --- | --- | --- | --- | --- |
| **MMD** | Spearman-Rho | 0.01 | 0.05 | -0.12 | -0.11 | 0.03 |
|  | p | 0.99 | 0.69 | 0.36 | 0.40 | 0.82 |
|  | n | 60 | 60 | 60 | 60 | 60 |
| **NRS** | Spearman-Rho | -0.11 | -0.02 | 0.03 | 0.25 | -0.22 |
|  | p | 0.40 | 0.89 | 0.81 | 0.40 | 0.10 |
|  | n | 60 | 60 | 60 | 60 | 60 |
| **Aura** | Spearman-Rho | -0.24 | 0.12 | -0.14 | -0.01 | -0.02 |
|  | p | 0.07 | 0.37 | 0.28 | 0.93 | 0.87 |
|  | n | 60 | 60 | 60 | 60 | 60 |

CGRP - Calcitonin gene-related peptide; E2 - estradiol, Tc - circadian testosterone; E2/P – estradiol-progesteron ratio

**Table 7 Correlations between CGRP, sex hormones and clinical parameters by aura status**

| **With Aura** |  | **CGRP** | **Tc** | **E2** | **Progesteron** | **E2/P** |
| --- | --- | --- | --- | --- | --- | --- |
| **MMD** | Spearman-Rho | -0.16 | 0.05 | -0.25 | -0.29 | 0.10 |
|  | p | 0.46 | 0.81 | 0.24 | 0.17 | 0.64 |
|  | n | 24 | 24 | 24 | 24 | 24 |
| **NRS** | Spearman-Rho | 0.03 | 0.08 | -0.08 | 0.09 | -0.11 |
|  | p | 0.89 | 0.69 | 0.70 | 0.69 | 0.63 |
|  | n | 24 | 24 | 24 | 24 | 24 |
| **Without Aura** |  | **CGRP** | **Tc** | **E2** | **Progesteron** | **E2/P** |
| **MMD** | Spearman-Rho | -0.15 | 0.12 | -0.09 | -0.01 | -0.07 |
|  | p | 0.93 | 0.51 | 0.58 | 0.95 | 0.69 |
|  | n | 24 | 24 | 24 | 24 | 24 |
| **NRS** | Spearman-Rho | -0.22 | -0.08 | 0.09 | 0.33 | -0.28 |
|  | p | 0.21 | 0.64 | 0.62 | 0.06 | 0.12 |
|  | n | 24 | 24 | 24 | 24 | 24 |
